# Underwater Imaging Using a 1 × 16 CMUT Linear Array

**DOI:** 10.3390/s16030312

**Published:** 2016-03-01

**Authors:** Rui Zhang, Wendong Zhang, Changde He, Yongmei Zhang, Jinlong Song, Chenyang Xue

**Affiliations:** 1 Key Laboratory of Instrumentation Science & Dynamic Measurement, North University of China, Ministry of Education, Taiyuan 030051, China; fly_zr@126.com (R.Z.); wdzhang@nuc.edu.cn (W.Z.); changde_henuc@163.com (C.H.); nucsong@163.com (J.S.); 2 Science and Technology on Electronic Test and Measurement Laboratory, North University of China, Taiyuan 030051, China; 3 School of Computer Science, North China University of Technology, Beijing 100144, China; zhangym@ncut.edu.cn

**Keywords:** capacitive micro-machined ultrasonic transducer linear array, transmission performance, synthetic aperture focusing technique, underwater imaging

## Abstract

A 1 × 16 capacitive micro-machined ultrasonic transducer linear array was designed, fabricated, and tested for underwater imaging in the low frequency range. The linear array was fabricated using Si-SOI bonding techniques. Underwater transmission performance was tested in a water tank, and the array has a resonant frequency of 700 kHz, with pressure amplitude 182 dB (μPa·m/V) at 1 m. The −3 dB main beam width of the designed dense linear array is approximately 5 degrees. Synthetic aperture focusing technique was applied to improve the resolution of reconstructed images, with promising results. Thus, the proposed array was shown to be suitable for underwater imaging applications.

## 1. Introduction

Ultrasound imaging has played an important role in various areas, such as medical diagnosis, medical treatment, nondestructive testing, and ultrasound microscopy [1,2,3]. The ultrasonic transducer is the core component of ultrasound imaging, and currently piezoelectric micro-machined ultrasonic transducers (PMUTs) based on the piezoelectric effect are widely used [4,5]. However, PMUT performance in underwater and medical applications is limited by material properties and impedance matching issues [2,6,7]. Capacitive micro-machined ultrasonic transducers (CMUTs) have many advantages over conventional PMUTs, such as wide bandwidth, high mechanical-electrical conversion efficiency, and ease of integration with electronic circuits to enhance signal-to-noise ratio [2,8,9,10,11,12]. Furthermore, CMUT membranes have low mechanical impedance, which makes them match well with air and other fluid media, and are suitable for manufacturing in large arrays [2,6]. These characteristics promote CMUTs as the development direction for next generation ultrasonic transducers.

Much research has been conducted regarding CMUT structural design, fabrication methods, and implementations. However, most studies have considered high frequency CMUTs (≥3 MHz) for medical imaging applications, with few studies on underwater imaging applications, which require low frequency. Roh *et al.* used finite element models to design a 1dimensional (1D) CMUT array robust to crosstalk [13]. ChiaHung *et al.* [14] designed and fabricated an underwater CMUT using full surface micro-machining technique. The transducer operated underwater at approximately 2 MHz with a detection range of 273 mm. Cheng *et al.* [15] realized B-mode imaging of a metal wire phantom using a 21-element 1-D array with 3.8 MHz central frequency and fractional bandwidth 116% in water, which limited the detection range. Doody *et al.* [16] designed a CMUT-in-CMOS array, which achieved central frequency 3.5 MHz, fractional bandwidth 32%–44%, and pressure amplitude 181–184 dB (μPa·m/V) at 15 mm when operated in a water tank. In this work, we designed and fabricated a 1 × 16 CMUT linear array with resonance frequency 700 kHz, and pressure amplitude 182 dB (μPa·m/V) at 1 m for use in underwater imaging.

## 2. Structural Design

**Figure 1:**
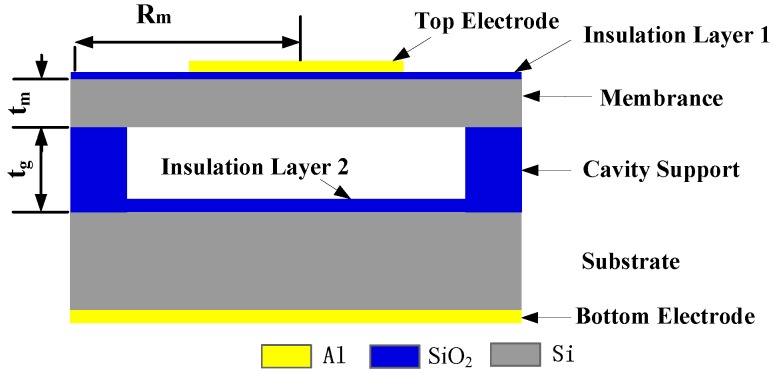
The structure of a cell.

CMUT array elements are composed of multiple sensitive cells connected in parallel. Each cell is composed of electrodes, vibrating membrane, vacuum cavity, insulating layer, and silicon substrate, as shown cross-sectional in Figure 1. A single CMUT array element often cannot meet imaging requirements with its low lateral resolution, low transmission power, and poor directivity. Therefore, a CMUT array composed of *N* identical elements is used to improve the imaging resolution.

The natural frequency is one of important performance parameters, whether operating in emission or receiving mode. When CMUTs work in the liquid, the resonant frequency of circular membrane $f_{r}$ is [17,18]:

(1) $$f_{r}=\frac{\frac{0.469t_{m}}{R_{m}^{2}}\sqrt{\frac{E}{\rho \left(1-\sigma^{2}\right)}}}{\sqrt{1+0.67\frac{\rho_{l}R_{m}}{\rho t_{m}}}}$$

where *E*, ρ, $\rho_{l}$, σ, $t_{m}$, $R_{m}$ represent the Young’s modulus, the density of membrane, the density of liquid, the Poisson’s ratio, the thickness of the membrane, and the radius of the membrane, respectively. High-resistivity silicon was chosen as the membrane material. From previous finite element software ANSYS analysis [19,20], following Equation (1), $t_{m}=3.5\;\mu m$ and $R_{m}=90\;\mu m$.

**Figure 2:**
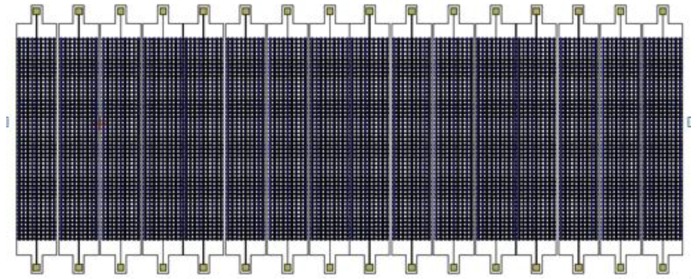
The resultant 1 × 16 CMUT array structure.

The emission performance of ultrasonic transducers is closely linked with its structural parameters. From previous studies [21], several directivity functions were deduced by Huygens’ Principle to guide the array structure design, and the resultant 1 × 16 CMUT structure is shown in Figure 2.

**Figure 3:**
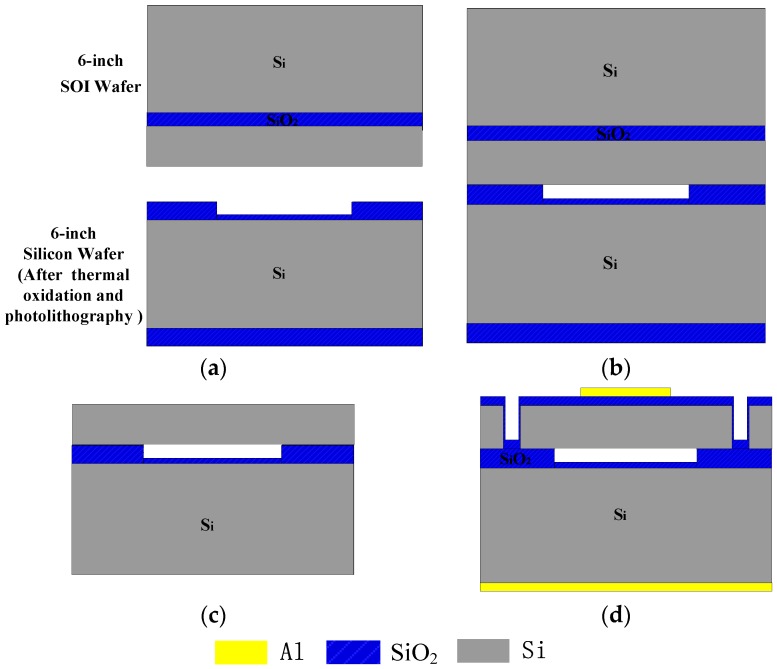
The main fabrication flow-charts. (**a**) part etched to form cavities; (**b**) bonding; (**c**) form membrane; (**d**) form isolation channel, insulation layer and electrodes.

Si-SOI low temperature wafer bonding technology [22,23] was used to fabricate the CMUT linear array (see Figure 3). The silicon wafer was first thermally oxidized, then part-etched to form cavities (t_g_ = 0.8 μm) and an insulation layer (0.15 μm) (Figure 3a). The silicon wafer and SOI wafer were bonded using low temperature bonding (Figure 3b), and the silicon substrate and buried oxide layer of the SOI wafer was eliminated (Figure 3c) to form the silicon membrane (t_m_ = 3.5 μm). Finally, an isolation channel was formed using photolithography and dry etching, and low pressure chemical vapor deposition was used to form a 0.15 μm insulation layer to prevent conductive contact between the top electrodes and the vibration membrane, and evaporation methods were used to form the top electrodes with Al. To ensure fine conductive contact between the bottom electrode and silicon substrate, the other side of the Si wafer had phosphorus ions implanted and metal Al deposited (Figure 3d).

## 3. Underwater Experimental

**Figure 4:**
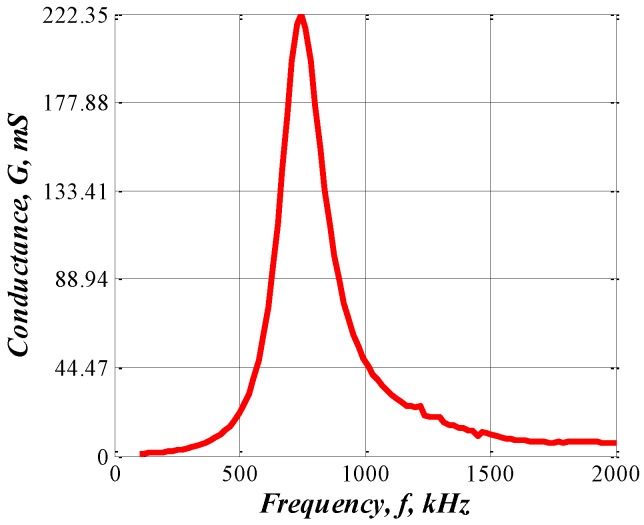
Resonant frequency.

The CMUT resonant frequency was found as shown in Figure 4. The linear array was encapsulated for insulation from the water [21], and a precision impedance analyzer (Agilent 4294A, Agilent Technologies, Santa Clara, CA, United States) was used to measure the array impedance in water (underwater penetration 0.45 m, water temperature 13 °C). The CMUT resonant frequency = 700 kHz, and electric conductance = 222.35 mS. The CMUT transmission characteristics (transmitting voltage response and directivity) were analyzed over the frequency range of interest (100–1000 kHz).

**Figure 5:**
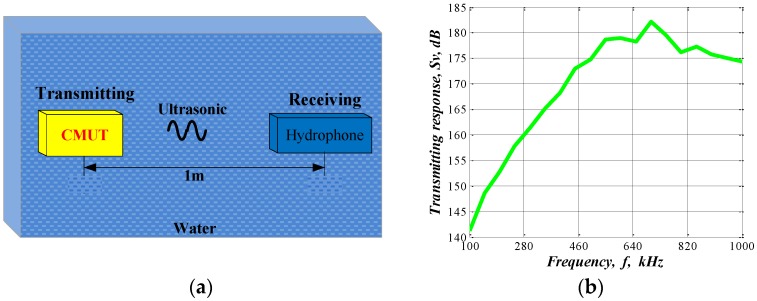
(**a**) Schematic diagram of the transmitting voltage response experiment; (**b**) Transmitting response.

The CMUT transmitting voltage response was measured as shown in Figure 5. A CMUT linear array (transmitter) and a standard hydrophone (receiver) were placed face to face, 1 m apart. The received electrical signal was displayed on an oscilloscope (Agilent 54624A), with direct current (DC) bias 20 V. The CMUT array was driven with a 5 cycle burst signal incorporating 100–1000 kHz and amplitude of 20 *V_pp_*.

The transmitting voltage response can be expressed as [24]:

(2) $$Sv=20lg\frac{u_{s}\cdot l}{u_{f}}-M_{o}$$

where *l* is the distance between the two transducers, $u_{f}$ is the applied driving voltage of the transmitting CMUT, $u_{s}$ is the collected voltage standard hydrophone, and $M_{o}$ is the receiving sensitivity of the hydrophone, the CMUT transmitting voltage response was obtained at different frequencies, as shown Figure 5b. The 1 × 16 CMUT linear array has resonant frequency 700 kHz, and pressure amplitude of 182 dB (μPa·m/V) at 1 m for underwater applications.

**Figure 6:**
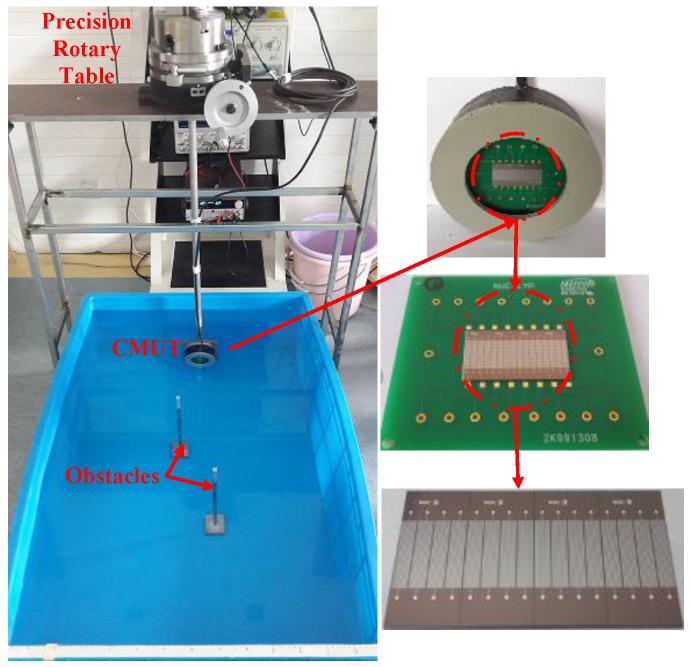
Sector scanning configuration.

The sector scanning experimental setup is shown in Figure 6. The operating conditions of the CMUT array were the same as previously, except it was now excited with a burst signal at 700 kHz for two cycles. The receiving and transmitting array was fixed on a precision rotary table and rotated to implement sector scanning for two-obstacle imaging.

**Figure 7:**
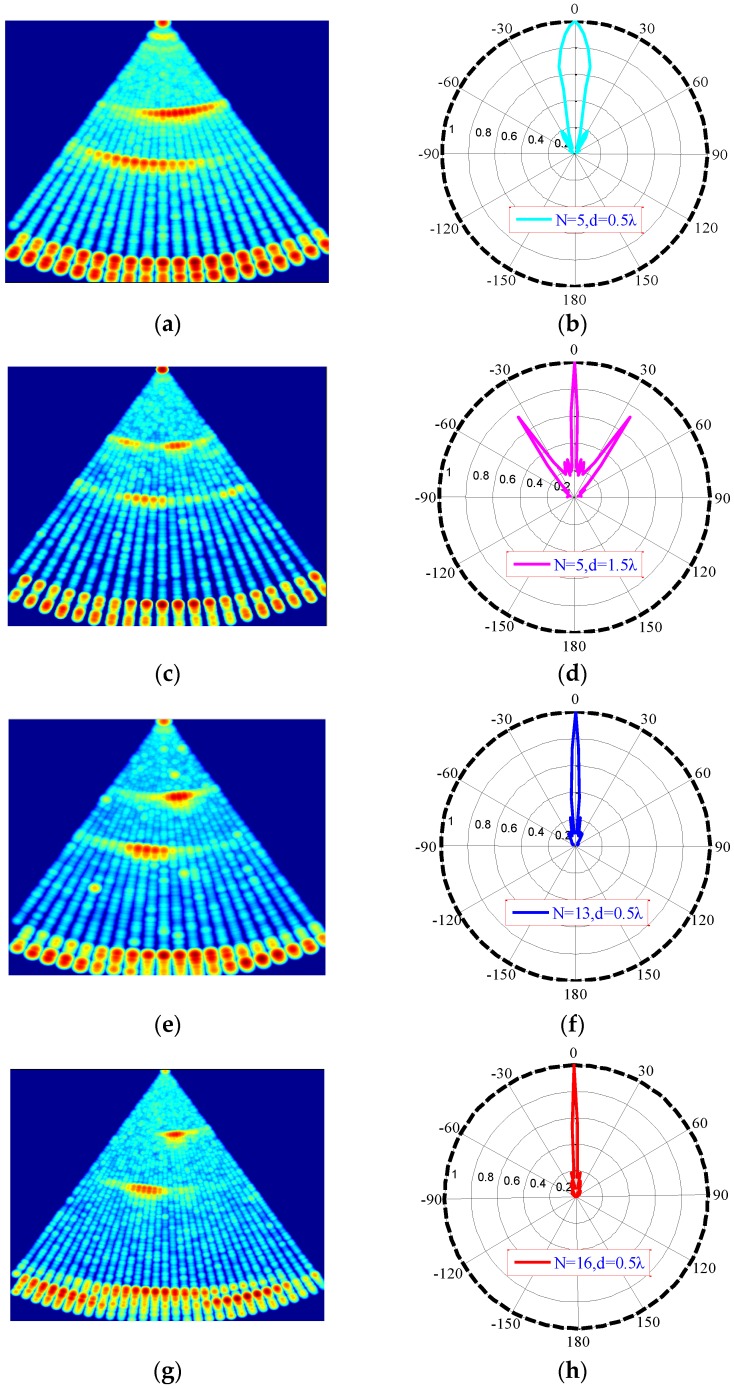
Sector scanning results: (**a**) N = 5, d = 0.5·λ; (**b**) directivity of diagram (a); (**c**) N = 5, d = 1.5·λ; (**d**) directivity of diagram (c); (**e**) N = 13, d = 0.5·λ; (**f**) directivity of diagram (e); (**g**) N = 16, d = 0.5·λ; (**h**) directivity of diagram (g).

Figure 7 shows the S-scan results and corresponding directivity diagrams for four transmitting conditions Figure 7b,d,f,h, where N is the number of array elements, d is the element spacing between array elements, and λ is the wavelength. The −3 dB main beam width of Figure 7b,d,f,h are approximately 21°, 6.4°, 8°, and 5°, respectively.

(1) For the fixed array element number (Figure 7a,c, or Figure 7b,d), the main lobe becomes sharper as *d* increases. However, when *d* > *λ* [19], strong grating lobes emerge, causing interference in the ultrasound image.
(2) For fixed array length (Figure 7c,e, or Figure 7d,f), the directivity of a dense array is better than that of a sparse array.
(3) For fixed element spacing (Figure 7a,e,g or Figure 7b,f,g), directivity improves as *N* increases. The wider main lobe also make severe interference.

Thus, *N* = 16, *d* = 0.5λ was selected as the transmission mode for subsequent underwater imaging.

## 4. Imaging

To reduce the influence of side lobes and improve the lateral resolution of reconstructed CMUT underwater images, the synthetic aperture focusing technique (SAFT) [25,26,27], an optimization method involving B-scan implemented by delay-and-sum on the received A-scan signals, was applied [28,29].

**Figure 8:**
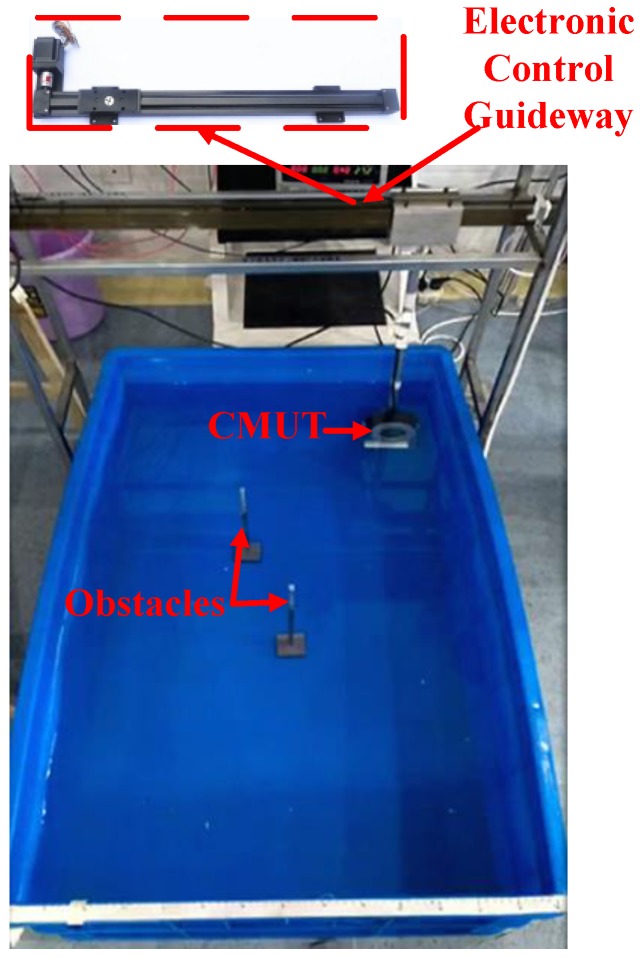
Linear scanning imaging configuration.

**Figure 9:**
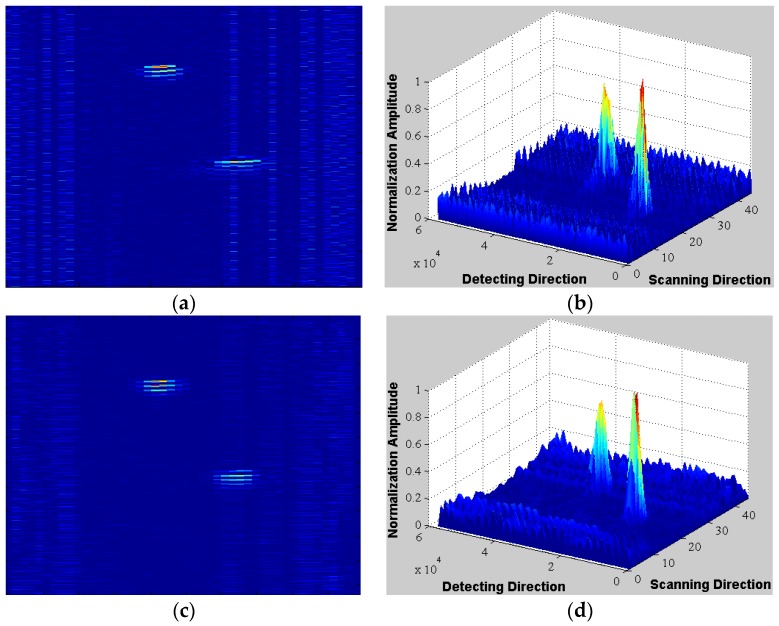
Reconstructed images (**a**) B-san reconstructed image; (**b**) 3-dimensional view of diagram (a); (**c**) SAFT reconstructed image; (**d**) 3 dimensional view of diagram (c).

Figure 8 shows the SAFT experimental setup. The operating conditions of the CMUT linear array were the same as for Section 3. The CMUT array was fixed on the electronic control guideway and moved horizontally to implement linear scanning for two-obstacle imaging. 16 elements were simultaneously excited. Figure 9 shows the SAFT reconstructed image (Figure 9a,b) is a significant improvement over traditional B-scan (Figure 9c,d), and artifacts caused by side lobes are effectively suppressed. Thus, the proposed array greatly reduces transmission issues without requiring phased transmission, thereby improving lateral resolution, which will be of great benefit in underwater imaging.

## 5. Conclusions

A 1 × 16 CMUT array was designed and fabricated for underwater imaging in the low frequency range. The transmission performance of the array was analyzed in a water tank, showing transmission voltage response 182 dB (μPa·m/V) was achieved at 1 m underwater. Directivity and sector scanning were also analyzed to determine the optimal transmission mode for linear underwater imaging. Significant resolution improvement was obtained by applying SAFT to the received A-scan signals. Thus, the proposed CMUT array shows great benefit for underwater detection applications, such as obstacle avoidance, distance measuring, and imaging.
